# Bioluminescence-based *in vitro* assay for rapid and quantitative anticoccidial screening

**DOI:** 10.3389/fcimb.2026.1773469

**Published:** 2026-02-26

**Authors:** Martina Felici, Callum de Hoest-Thompson, Benedetta Tugnoli, Ester Grilli, Virginia Marugan-Hernandez

**Affiliations:** 1 Vetagro S.p.A., Reggio Emilia, Italy; 2 Department of Pathobiology and Population Sciences, Royal Veterinary College, University of London, Hatfield, United Kingdom; 3 Dipartimento di Scienze Mediche Veterinarie (DIMEVET), Università di Bologna, Bologna, Italy; 4 Vetagro Inc., Miami, FL, United States

**Keywords:** anticoccidials, bioluminescence assay, botanicals, *Eimeria tenella*, NanoLuc luciferase

## Abstract

Coccidiosis, caused by *Eimeria* parasites, is a major threat to global poultry production, and increasing restrictions on conventional anticoccidial drugs highlight the need for safer, more sustainable alternatives. Progress has been hindered by the lack of rapid, sensitive, and animal-sparing *in vitro* assays for quantifying parasite replication and drug efficacy. This study reports the development of a novel bioluminescent platform for anticoccidial screening based on a genetically modified *Eimeria tenella* line expressing NanoLuc luciferase (EtNluc). Parasite-associated bioluminescence enabled rapid and quantitative monitoring of intracellular development, allowing the tracking of different replication phases through schizont formation and merozoite release. Time course analysis showed minimal changes in relative light units (RLU) between 2 and 24 hours post infection (hpi), followed by a marked increase between 24 and 72 hpi, consistent with parasite replication. Among the tested multiplicities of infection (MOI), 4:1 exhibited the fastest growth, described by a linear model (slope = 2908 RLU/h, R^2^ = 0.84). A same-well repeated-measure analysis (2 and 72 hpi) confirmed the dose-dependent replication, with mean slopes of 2052.85, 765.07 and 523.63 RLU/h, respectively, supporting the selection of the MOI 4:1 for anticoccidial screening. These experimental conditions were used to evaluate the anticoccidial efficacy of commercial anticoccidial drugs (salinomycin and robenidine) and natural compounds (thyme and oregano essential oils, thymol, and carvacrol) under two experimental designs: short pre-incubation of sporozoites, and continuous exposure throughout intracellular development. Pre-incubation with commercial anticoccidials reduced invasion approximately to 65% for salinomycin and 44% for robenidine, whereas the essential oils and their bioactive constituents inhibited invasion by 30-55%, and reduced the replication slope to 33-60% of control values. Continuous exposure significantly impaired intracellular development for all treatments, reducing replication to 10-30% of controls, providing additional evidence that plant-derived compounds can complement commercial anticoccidials for integrated strategy for coccidiosis control in chickens. Overall, the EtNluc bioluminescent system provided a rapid, sensitive, and scalable method for quantifying *E. tenella* growth, suitable for *in vitro* anticoccidial screening, supporting the characterization of novel anticoccidial while reducing reliance on animal experimentation.

## 1 Introduction

Coccidiosis, caused by protozoan parasites of the genus *Eimeria*, is one of the most economically significant diseases affecting poultry worldwide. Among them, *Eimeria tenella* is recognized as one of the most pathogenic species, responsible for severe lesions in the ceca, that eventually lead to reduced weight gain and deaths in chickens. Furthermore, its suitability for *in vitro* cultivation makes it a valuable model organism for the evaluation of anticoccidial compounds (Ball et al., 1990). The economic burden of this disease is substantial, estimated at 13 billion US dollars per year, due to direct losses in poultry production and costs associated with prevention and treatment (Blake et al., 2020).

The control of coccidiosis in broilers relies on the use of ionophores and chemical anticoccidials, however, the spreading of resistance to these treatments has emphasized the urgency of improving research on alternative therapeutic strategies (Noack et al., 2019). In this context, botanicals have gained increasing attention as promising alternatives due to their natural origin, diverse composition, and potential to reduce parasite load while supporting gut health (Muthamilselvan et al., 2016; Noack et al., 2019). These compounds comprehend various classes of molecules and their efficacy can vary depending on various factors, so the assessment of their efficacy needs continuous screening (Felici et al., 2024).

In recent years, anticoccidial research has seen important advances in the development of rapid, sensitive, and well-standardized *in vitro* methods to quantify parasite invasion and proliferation, with several new approaches now available. Nevertheless, conventional techniques based on *in vivo* experimentation, such as oocyst counting, histological analysis, or quantitative PCR (qPCR) remain widely used. These methods, while informative, can still be time-consuming, labor-intensive, and in some cases require animal sacrifice, with assay-to-assay variability arising from long sample processing (Felici et al., 2021). Evaluation of alternative anticoccidial compounds through *in vitro* approaches could allow high throughput screening, reducing the use of animals and related costs. Current *in vitro* methods rely on the use of microscopy-based assays or quantification of the intracellular parasite DNA by qPCR (Jenkins et al., 2014; Thabet et al., 2015; Marugan-Hernandez et al., 2020; Felici et al., 2021).

While sporozoite staining and manual counting have long been employed to estimate anticoccidial efficacy (Burt et al., 2013; Felici et al., 2020), these approaches are intrinsically subjective and prone to operator bias, therefore, automated systems for quantification offer a more accurate and reproducible method to assess treatments (Marugan-Hernandez et al., 2020). Stably-transfected strains of *E. tenella* expressing reporter genes (*i.e.* fluorescent proteins) have been used to track the development of the parasite in cell culture. For instance, Bussiére et al. (2018) (Bussière et al., 2018) used a recombinant strain expressing the yellow-fluorescent protein (YFP) under the *mic1* promoter to visualize parasites in invasion and schizont development assays, while another strain was created by transfecting *E. tenella* with the *mCherry* gene under the *actin* promoter to facilitate visualization of sexual stages of the parasite in cell culture (Bussière et al., 2018). Later, Marugan-Hernandez et al. (2020) (Marugan-Hernandez et al., 2020) used *E. tenella* YFPmYFP (EtdYFP) (Clark et al., 2008) and a custom ImageJ tool to perform a semi-automatic quantification of the parasite development in cells, correlating the data to qPCR analysis. This method was also used to screen anticoccidial activity *in vitro* and subsequently to evaluate natural compounds, validating its applicability to evaluate the anticoccidial effects of botanical compounds (Felici et al., 2024).

Further innovations in drug screening include the use of bioluminescence systems, as these offer a dynamic method to quantify viability and replication, and has been increasingly applied to develop *in vitro* models for infectious diseases (Azevedo et al., 2014; Vinayak et al., 2015; England et al., 2016; Berry et al., 2018; Smith et al., 2023). Among the various luciferases available, NanoLuc (NLuc) has gained increasing attention due to its small size, high stability, and exceptional luminescent signal intensity with minimal background noise (England et al., 2016). NLuc is an artificial coelenterazine-dependent luciferase generated from the organism *Oplophorum gracilirostris*. Its long-lasting bright bioluminescence triggered with the synthetic substrate furimazine, has made this enzyme popular as a reporter in a variety of analytical systems (England et al., 2016). NLuc-based systems have been successfully applied in several protozoan models, enabling high-throughput screening and real-time monitoring of infection dynamics *in vitro*. For example, Azevedo et al. (2014) produced a luminescent strain of *Plasmodium falciparium*, causative parasite of malaria, using Nluc to create sensitive and affordable growth assays to understand the mode of action of antimalarian drugs (Azevedo et al., 2014). A similar approach was adopted by Vinayak et al. (2015), who optimized a Nluc luminescent strain of *Cryptosporidium parvum* to evaluate the basis of drug susceptibility by gene knock out *in vitro* (Vinayak et al., 2015). Additionally, other research studying *Toxoplasma gondii*, and *Leishmania mexicana* has used modified approaches of Nluc assays to evaluate parasite viability in an *in vitro* cell model during drug testing (Berry et al., 2018; Smith et al., 2023).

In this study, the generation of a genetically modified *E. tenella* population stably expressing NLuc (EtNLuc) is reported, with a focus on development for rapid screening of potential anticoccidial compounds. This transgenic parasite population enabled robust luminescent detection of intracellular parasite stages in cultured cells. This system also provided a highly sensitive, rapid, and reproducible method to quantify *E. tenella* invasion and replication *in vitro*, suitable to perform anticoccidial compound screenings. The development of this luminescence-based platform represents a valuable advancement in the field of coccidiosis research, reducing the number of animals used for the generation of oocysts, and contributing to more efficient discovery pipelines and screening accuracy.

## 2 Materials and methods

### 2.1 Parasites and animals

Oocysts of *Eimeria tenella* Wisconsin (EtWis) reference strain or EtNluc transgenic population (described below) were propagated in four-week-old Lohman VALO White Leghorn chickens, supplied by the Animal and Plant Health Agency (APHA, UK) and raised and maintained in specific pathogen free conditions at the Royal Veterinary College (RVC, UK) following standard procedures (Pastor-Fernández et al., 2019).

### 2.2 Plasmid construct and generation of an *E. tenella* bioluminescence population

*Nluc* gene sequence was amplified by PCR using Platinum Taq (Invitrogen) from the plasmid pNL1.1 (kindly provided by Dr Sateriale) (Vinayak et al., 2015) using specific primers (FwNluc: TGTATTCACATTCAAAATGTCGGTATTTACATTGGAAGATGCGTTC). The amplicon was cloned using Gibson assembly (NEB) into the plasmid p5M2_mChe (containing a *5’EtMIC1-mCitrine* and a *5’EtMIC2-mCherry* cassette) (Marugan-Hernandez et al., 2017), previously linearized with XbaI and BglII (to release and replace the *mCherry* coding sequence). The correct *Nluc* sequence was confirmed by sequencing (Eurofins) using CLC Main Workbench (Qiagen). The new plasmid (p5M2_Nluc) was amplified using a Midi Prep Kit (Qiagen) and digested with PsiI for linearization prior transfection into *E. tenella* sporozoites.

Standard protocols for sporozoite excystation from oocysts were followed (Pastor-Fernández et al., 2019). Transient transfection of p5M2_Nluc into freshly purified sporozoites was performed using Nucleofector 4D (Lonza) following well-stablished protocols (Pastor-Fernández et al., 2019). Successful transfection was confirmed by observing mCitrine-expressing sporozoites using a Leica DMI3000B fluorescent microscope. A stable *E. tenella* bioluminescent population (EtNluc) was obtained after four consecutive rounds of chicken infections with mCitrine-fluorescent parasites (oocysts or sporocysts) selected by Fluorescence-activated cell sorting (FACS; BD AriaFusionTM) (Pastor-Fernández et al., 2019).

### 2.3 Cell culture

Madin-Darby bovine kidney (MDBK) NBL-1 line was used to sustain sporozoites infections. Cells were maintained in Advanced Dulbecco’s Modified Eagle Medium (AdDMEM; Gibco) supplemented with 5% FBS (Sigma), 100 U/ml penicillin/streptomycin (Fisher) and 5 ml of GlutaMAXTM (Gibco). MDBK cells were passed twice weekly following standard procedures (Marugan-Hernandez et al., 2020). Two hours prior sporozoite infection, cells were plated at 0.05 million/well in CorningTM 96-well white/clear bottom plates (Fisher).

### 2.4 Measuring luminescence

Twenty-five microliters of Nano-Glo^®^ Live Cell Reagent (Promega) were added per well to freshly purified sporozoites or cell monolayers infected with sporozoites of the EtNluc population prepared in 100 µl of supplemented AdDMEM in CorningTM 96-well white/clear bottom plates (Fisher). Luminescence was measured immediately after adding the reagent using a Tecan Infinite 200Pro (Mode: Luminescence; Attenuation: None; Integration time: 1000 ms) and expressed as relative light units (RLU).

### 2.5 Optimization of cultivation ratios for the bioluminescence-based assay

Freshly hatched sporozoites were purified following the methods described previously (Pastor-Fernández et al., 2019), and used to infect cell monolayers (prepared at 0.05 million/well) in 96-well plates at the following concentrations: 0.05, 0.1, 0.2 million/well, corresponding to a multiplicity of infection (MOI) of 1:1, 2:1 and 4:1 respectively. At 2 hours post invasion (hpi), extracellular sporozoites were removed from the wells, and the medium was replenished. The cells were left in culture up to 72 hpi to track the parasite’s development until the generation of mature schizonts and first generation merozoites. Three wells were used per condition, and two biological replicates were performed for each set of experiments.

To evaluate the luminescence-based detection of intracellular sporozoites and schizont replication, RLU were measured at four different time points: 2, 24, 48 and 72 hpi, as single independent measurements (SIM). For each time point, independent plates were used to avoid repeated washing or handling that could interfere with the experiment. The three experimental ratios were evaluated by comparing the linear regression curves drawn within 24 hpi and 72 hpi, corresponding to the nuclear replication phase (Marugan-Hernandez et al., 2020).

Subsequently, a second set of experiments was performed using a single plate with repeated measurements done at 2 and 72 hpi (RM). This approach was adopted to evaluate the suitability of using the same samples for consecutive measures, assessing whether the repeated medium changes and measurements affect the luminescence signal (RLU) compromising the overall assay consistency.

The efficiency of invasion was quantified as RLU measured in the infected samples after subtraction of background signal detected in uninfected MDBK cells. Parasite replication was determined by calculating the slope of the linear regression curve between the initial and the last time point, as previously described (Arias-Maroto et al., 2022).

### 2.6 Anticoccidials and compounds preparation

Commercial anticoccidials: salinomycin (Sigma-Aldrich), derived from *Streptomyces albus*, was dissolved in ethanol, while robenidine hydrochloride (Sigma-Aldrich) was dissolved in dimethyl sulfoxide. The compounds were used at final concentrations of 1 ppm and 5 ppm, respectively.

Essential oils and nature identical compounds were tested at a concentration of 40 ppm and 20 ppm, respectively, based on previous studies (Felici et al., 2023; Felici et al., 2024). Oregano essential oil (OEO) was provided by Galen-N and thyme essential oil (TEO) from Grupo Indukern. Thymol (THY) and carvacrol (CAR) were all purchased analytical grade from Sigma-Aldrich. All stock solutions were prepared in ethanol and added to cells in supplemented AdDMEM medium; in all cases the final concentration of ethanol was below 1000 ppm, corresponding to less than 0.1% (v/v).

### 2.7 *In vitro* bioluminescence assay for anticoccidial compound screening

For the anticoccidial compounds’ testing, MOI 4:1 was selected as dose of infection. Four wells were used per condition, and two biological replicates were performed for each set of experiments.

Pre-incubation exposure: freshly hatched sporozoites were incubated for 1 h with the essential oils, nature identical compounds, anticoccidial, or left untreated (control). Afterwards, they were added to the cell monolayers. After 2 h, the infected cells were washed to remove extracellular sporozoites and fresh cell medium, with no treatment, was replenished.

Continuous exposure: sporozoites were mixed with treatments and added directly to the cell monolayers. After 2 h, the cells were washed to remove extracellular sporozoites, and the cell medium with the same treatments was replenished. In this case the treatments were not stopped until the end of the experiment.

Invasion efficiency for each treatment was calculated with the following equation by Thabet et al. (2017) (Thabet et al., 2017) modified as:


$$\%\;of\;invasion=100\;\times \;\left(1-\;\frac{RLU\;in\;treated\;sample}{RLU\;in\;control}\right)$$

Comparison of replication rates after drug treatment were done by calculating normalized slopes of the growth curve between 2 hpi and 72 hpi, using the previously reported formula by Arias-Maroto et al. (2024) (Arias-Maroto et al., 2022) which was adapted as follows:


$$\%\;slope\;=\;100\;\times \frac{{\left[\frac{RLU\;\left(72\;hpi\right)-RLU\;\left(2hpi\right)}{70\;\left(time\;lag\right)}\right]}_{treated\;sample}}{{\left[\frac{RLU\;\left(72\;hpi\right)-RLU\;\left(2hpi\right)}{70\;\left(time\;lag\right)}\right]}_{control}}$$

### 2.8 Statistical analysis

Statistical analyses and data visualization were performed using GraphPad Prism (version 10.2.3). Background-corrected RLU values were averaged across three replicates of two independent experiments for each time point and MOI and they were displayed with standard error of means (SEM). For SIM experiment a linear regression model was fitted to the mean values within the 24 and 72 hpi, and model adequacy was evaluated based on the coefficient of determination (R²). For SIM and RM experiments, two-way ANOVA was applied to estimate differences among samples, followed by Tukey’s multiple comparison test to identify significant differences between MOI within the same time point.

For the anticoccidials assays, the normalized invasion efficiency and replication slope values were averaged across four replicates within each independent experiment. Differences among groups were assessed using a one-way ANOVA, followed by Tukey’s multiple comparison test for *post hoc* analysis. Statistical significance was defined as p ≤ 0.05.

## 3 Results

### 3.1 Generation of a bioluminescence population derived from *E. tenella* Wisconsin (EtWis) reference strain

The *Nluc* gene was cloned downstream the *microneme 2* promoter region of *E. tenella* within the pCIT backbone plasmid expressing the fluorescent marker mCitrine (Marugan-Hernandez et al., 2017). Plasmids were transfected to sporozoites of EtWis and these were used to generate a transgenic population thought successive passages in chickens and FACS sorting. After four rounds of amplification, a stable population, EtNLuc (>90% fluorescence, **Supplementary Figure 1**) was obtained. Bioluminescence emission was confirmed in freshly hatched EtNluc sporozoites by comparing these with sporozoites from the parental strain EtWis (**Supplementary Table 1**). Bioluminescence measurements of infected monolayers with EtWis represented only an average value below 0.5% of the RLU values obtained with EtNLuc, validating the successful expression of Nluc in sporozoites of this population.

**Figure 1:**
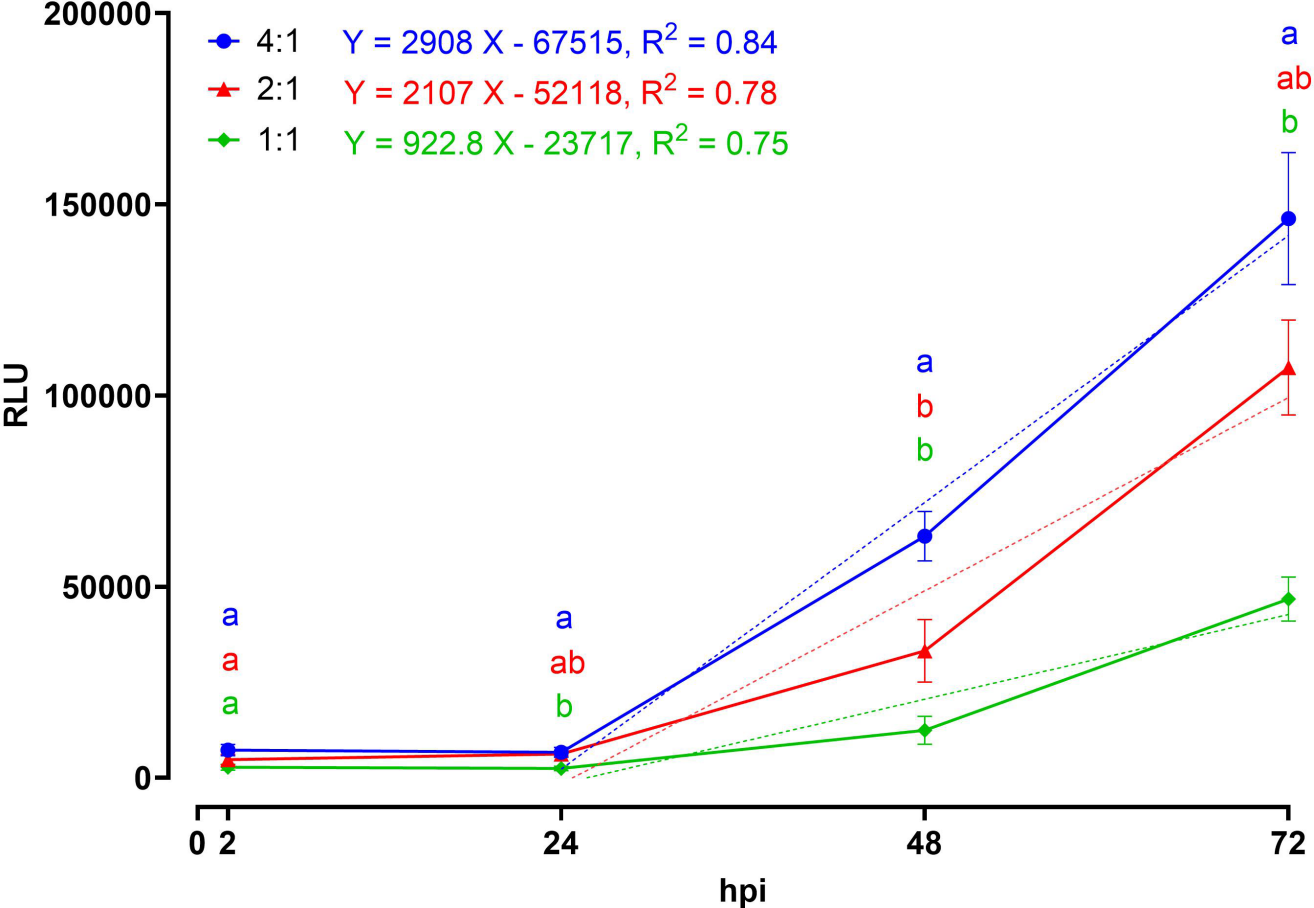
Graph showing the intracellular replication of EtNLuc with different multiplicities of infection (MOI 4:1; 2:1; 1:1) expressed in relative light units (RLU) over time. The dotted line corresponds to the respective linear interpolation within 24 and 72 hpi. Different letters indicate significant differences among the different doses of infection within the same time point assessed with two-way ANOVA and *post-hoc* Tukey’s multiple comparison test (n=6, p ≤ 0.05). The simple linear regression line equations and R^2^ values are reported next to the relative MOI following the color code: blue = MOI 4:1, red = MOI 2:1, green = MOI 1:1.

The use of bioluminescence parasites (EtNLuc) enabled reliable detection of parasite multiplication on the SIM experiment, performing single RLU reading per well. The temporal dynamics of the response differed markedly according to the infecting dose (**Figure 1**). At 2 and 24 hpi, prior nuclear replication, all doses showed comparable values, with no clear separation between ratios. From 48 hpi onward, a clear dose-dependent divergence became evident: the 4:1 treatment displayed the highest increase in RLU, the 2:1 reached intermediate levels and the 1:1 remained consistently lower. Linear regression confirmed these trends (**Figure 1**), with slopes decreasing from 2,908 at the highest dose of infection, to 2,107 at the intermediate one, and 922.8 at the lowest MOI, indicating that both the onset and the rate of increase were accelerated under higher infection doses. The statistical comparison further supported these findings, showing significant differences among doses from 48 hpi onward, with the separation becoming more marked at 72 hpi. Notably, the goodness of fit improved with increasing MOI, with R^2^ values of 0.84, 0.78 and 0.75 for MOI 4:1, 2:1, and 1:1, respectively.

### 3.2 Optimization of the bioluminescence -based assay for anticoccidial screening

**Figure 2:**
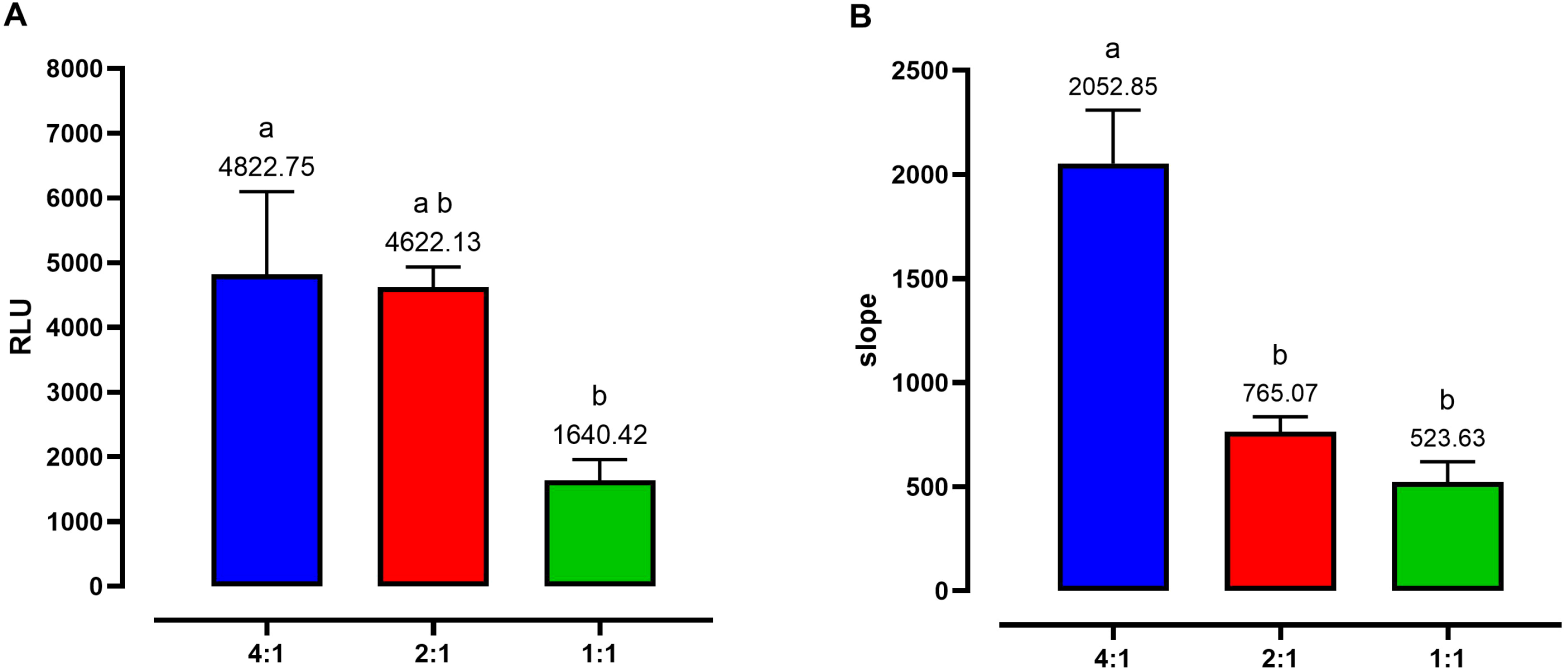
Graphs showing the invasion and intracellular replication of EtNLuc applying the repeated measurements protocol (RM). **(A)** represents the invasion at 2 hpi expressed as relative luminescence units (RLU). **(B)** represents the replication of the parasite expressed as the slope of the replication curve between 2 and 72 hpi. Different letters indicate significant differences among the different doses of infection evaluated by one-way ANOVA and *post-hoc* Tukey’s multiple comparison test (n=6, p ≤ 0.05).

The intracellular growth was further assessed with the protocol RM, performing repeated RLU readings at 2 and 72 hpi in the same wells (**Figure 2**). Consistently with the SIM protocol, all the different MOI allowed the detection of parasite replication. The highest level was reached by the 4:1 dose, followed by the others in a dose-dependent manner (**Figure 2**). Replication of the parasite was expressed as the slope of the linear regression curve calculated within the timeframe of 2 to 72 hpi. The highest level of expression was recorded for MOI 4:1, with an average slope value of 2,052.85 (**Figure 2**). The other two infection doses led to a similar level of development, with values of 765.07 for MOI 2:1 and 523.63 for MOI 1:1 (**Figure 2**).

### 3.3 Anticoccidial efficacy of natural compounds following pre-incubation exposure

**Figure 3:**
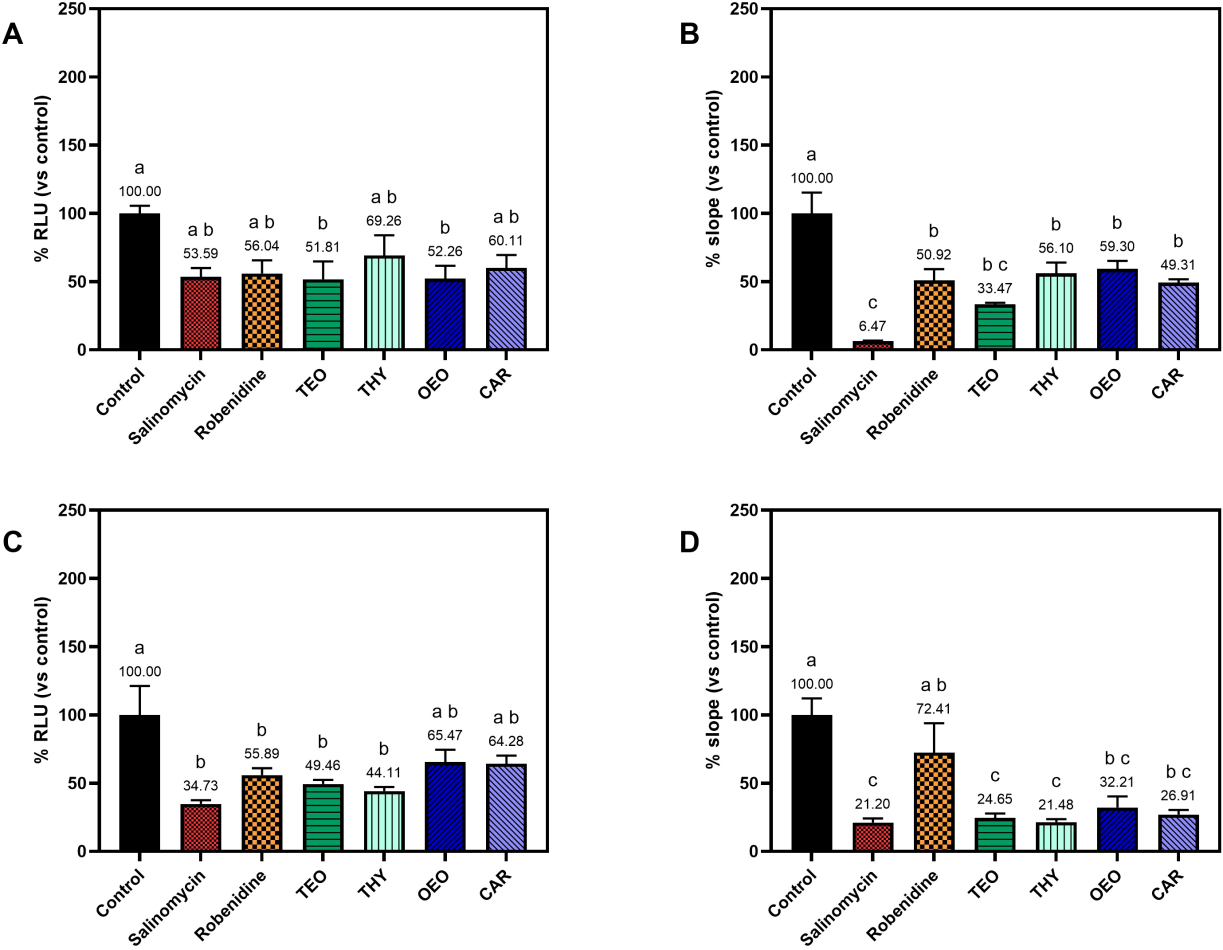
Graph showing the invasion and intracellular replication of EtNLuc following pre-incubation exposure with anticoccidials and natural compounds. Invasion is expressed as relative light units (RLU) normalized to the untreated control at 2 hpi [**(A)** for experimental replicate 1 and **(C)** for experimental replicate 2]. Parasite replication is expressed as the normalized slope of the growth curve measured of each treatment [**(B)** for ER1 and **(D)** for ER2]. Different letters indicate significant differences among the different groups assessed with one-way ANOVA and *post-hoc* Tukey’s multiple comparison test (n=4, p ≤ 0.05).

Treatment with anticoccidial drugs and natural compounds influenced invasion and replication after pre-incubation for 1 h prior invasion (**Figure 3**). At 2 hpi, invasion was affected by almost all the treatments. The two commercial anticoccidials significantly reduced invasion in the experimental replicate 2 (ER2) by 65.27% with salinomycin and 44.11% for robenidine, whereas in experimental replicate 1 (ER1) the efficacies showed a reduction, however, they were not significantly different from the control. A similar behavior was observed for the natural compounds: TEO was one of the most effective treatments, with significant invasion inhibitions ranging between 42.19% (ER1) and 50.54% (ER2). The other natural treatments had intermediate efficacy, ranging between 30.74% and 55.89% for THY, 34.53% and 47.74% for OEO, 35.72% and 38.89% for CAR (**Figures 3A, C**).

Parasite replication was measured as the slope of the growth curve measured within the two time points. In both experimental replicates the highest slope was observed on the control, meaning that replication was also affected by the treatments (**Figures 3B, D**). In ER1, salinomycin was the most effective (% slope vs. control = 6.47), while robenidine’s value was higher (% slope vs. control = 50.92), but still significantly lower than the control. Regarding the natural compounds, all of them were significantly lower than the control, with TEO being the most effective (% slope vs. control = 33.47); THY, OEO and CAR slopes were slightly higher, ranging between 49.31% and 59.30%. In ER2, despite the presence of overall lower values, a similar tendency was observed. Salinomycin was confirmed as the most effective on replication, with a % slope vs. control of 21.20. Robenidine reached values similar to the untreated control while all the natural compounds were significantly different, with % slope values ranging between 21.48 (THY) and 32.21 (OEO).

### 3.4 Anticoccidial efficacy of natural compounds following continuous exposure

**Figure 4:**
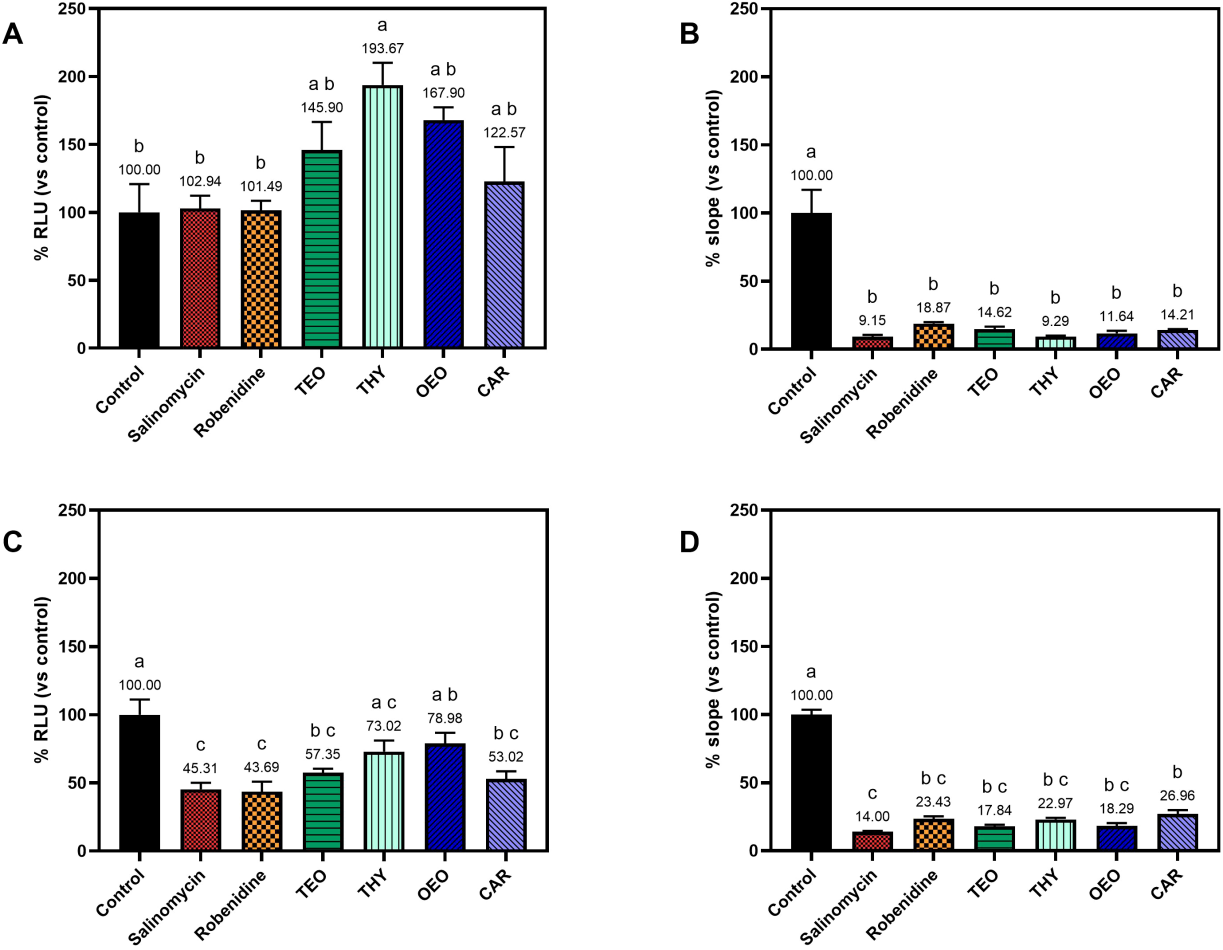
Graph showing the invasion and intracellular replication of EtNLuc following continuous exposure to anticoccidials and natural compounds. Invasion is expressed as relative light units (RLU) normalized on the untreated control at 2 hpi [**(A)** for experimental replicate 1 and **(C)** for experimental replicate 2]. The replication is expressed as the normalized slope of the growth curve measured of each treatment [**(B)** for ER1 and **(D)** for ER2]. Different letters indicate significant differences among the different groups assessed with one-way ANOVA and *post-hoc* Tukey’s multiple comparison test (n=4, p ≤ 0.05).

Treatment of infected MDBK cells monolayers with the selected compounds revealed notable effects on invasion and replication when the treatment was continuously applied, from the time of infection (0 hpi) until the end of the experiment (72 hpi) (**Figure 4**). In ER1, no decrease in invasion was observed for any of the treatment (**Figure 4A**); whereas in ER2, a marked reduction of intracellular sporozoites was observed for the commercial anticoccidial drugs (54.69 and 56.31 for salinomycin and robenidine, respectively), TEO and CAR (42.65% and 46.98%, respectively) (**Figure 4C**). A slight but not significant reduction of invasion of 26.98% and 21.02% was also observed for THY and OEO, respectively.

Parasite replication was consistently impaired across both experiments (**Figures 4B, D**). All treatments significantly reduced intracellular development. Salinomycin showed the strongest effect (11.58% average slope), while robenidine was slightly less effective than the other anticoccidials. Essential oils also reduced replication, with slopes ranging from 11.64% to 18.29%. THY and CAR yielded higher slopes (16.12–20.58%) but remained significantly lower than the controls.

## 4 Discussion

This study describes the generation of a transgenic population, EtNLuc, derived from the reference strain EtWis, that incorporates both fluorescent and bioluminescent reporter cassettes. To verify whether the bioluminescence produced by Nluc was suitable to detect replication, the parasite was taken through a time course assay with luminescence measured at 2, 24, 48, 72 hpi. The increase of signal detected over time was comparable to observations from previous work using quantification by qPCR or fluorescence measurements using the EtdYFP strain (Marugan-Hernandez et al., 2020; Arias-Maroto et al., 2022). Between 2 and 24 hpi there was no significant change in the levels of luminescence, consistent with the qPCR data using an equivalent infection set up (Arias-Maroto et al., 2022). This period is representative of sporozoites rounding up forming a trophozoite, without the nuclear replication occurring (Pacheco et al., 1975; Marugan-Hernandez et al., 2020). Between 24 and 48 hpi there was a significant increase in luminescence readings, consistent with the increase in genome copy number previously detected by qPCR, representing the development from trophozoites into immature schizonts and subsequent nuclear replication (Pacheco et al., 1975; Marugan-Hernandez et al., 2020). Between 48 and 72 hpi the signal kept increasing, correlating with the increasing genome copy numbers described between 44 and 52 hpi by Arias-Maroto et al. (2022) (Arias-Maroto et al., 2022). These time points correspond to the period of development representing maturing schizonts and the release of merozoites (Pacheco et al., 1975; Marugan-Hernandez et al., 2020). Thus, the new EtNLuc bioluminescent strains reliably reflect parasite replication at the selected time points corresponding to specific developmental processes.

Previous studies have shown that the 4:1 parasite to cell infection ratio allowed the supported a higher number of infected cells on the monolayer in comparison to 2:1 or 1:1 ratios (73%, 60% and 39% respectively) (Arias-Maroto et al., 2022), however, how these initial proportions affect replication rates has not been studied before. At 2 hpi and 24 hpi, luminescence readings were not significantly different between the three MOIs, however, a clear trend of lower reading at lower MOI was observed. At 48 hpi a significant increase was observed between 4:1 and the other MOIs. Similarly, at 72hpi, an analogous pattern was observed. The comparison of growth curves for each ratio showed that lower number of parasites led to a slower replication rate (slope), supporting a direct relationship between initial number of intracellular sporozoites and their replication rates. Since the primary aim of the EtNluc strain is to be used for the assessment of potential anticoccidial therapies, the MOI 4:1 was selected as this provided a wider dynamic range to evaluate inhibitory effects in parasite replication. Additionally, the higher goodness of fit value (R^2^) observed at a MOI 4:1, indicates that this dose of infection not only produced the highest signal, but also the most predictable and reproducible growth kinetics, making it the optimal condition for quantitative parasite proliferation assays.

The initial time course experiment for the bioluminescence-based assay standardization discussed above was performed by doing single readings per plate. To evaluate the impact of performing continuous measurements on the same infected monolayer, only 2 and 72 hpi points were selected, as based on the single readings, they would provide enough significance for studies evaluating inhibition of invasion and replication. The same three MOIs were evaluated, showing equivalent significance and trends that in the single measurement experiments. This supported the adaptability of the method to repeated reading over the same plate. This repeated measurement system reduces the need of oocysts for each tested compound and, consequently, the number of experimental chickens needed to produce these oocysts, in accordance with the 3R (Replacement, Reduction and Refinement) principle of animal experimentation (Russel and Burch, 1959).

When comparing the results obtained from the EtNluc strain to those generated by the qPCR method using EtWis parasites, as well as imaging with the fluorescent EtdYFP strain, the outcomes were comparable (Marugan-Hernandez et al., 2020; Arias-Maroto et al., 2022). This highlights the bioluminescence-based assay as a valuable and reliable tool. While fluorescent reporters are ideal to track the presence and localization of parasites in biological samples and cell culture, they still present certain limitations. For instance, their moderate sensitivity can be hindered by sample autofluorescence, and their fluorescence signal is prone to photobleaching over time. Moreover, proper visualization often requires cell fixation with paraformaldehyde and washing steps, which restricts the use of these methods for real-time or live-cell quantification (Haugwitz et al., 2008). Hence, the bioluminescence-based system is less labour-intensive, requires fewer steps, and produces results more quickly, enabling the simultaneous screening of a larger number of compounds.

The NLuc reporter expressed by EtNLuc sporozoites provided stable luminescence, enabling sensitive detection of intracellular parasites during treatment with natural compounds and anticoccidial drugs. Using this reporter system, the efficacy of anticoccidials and botanical compounds was evaluated in two distinct assay formats: a pre-incubation protocol, in which sporozoites were exposed to compounds for 1 h prior to invasion and during the first 2 hpi; and a continuous exposure protocol, in which compounds remained present throughout intracellular development, form the addition of sporozoites to the cell monolayers until 72 hpi. In the first assay, the experiment was designed to evaluate specific effects while the parasites remain extracellular; whereas in the second protocol, the potential intracellular activity of the compounds was assessed by maintaining the treatment after the invasion of the parasite.

In both assays, salinomycin and robenidine significantly impaired parasite invasion. Salinomycin consistently achieved higher inhibition, in line with its well-documented ability to disrupt ionic gradients and compromise sporozoite viability prior to cell entry (Noack et al., 2019). The short pre-treatment was also sufficient to significantly inhibit intracellular replication, indicating a lasting effect of the molecule. On the other hand, robenidine showed a different pattern of efficacy. Robenidine is a guanidine derivative whose main action is the inhibition of maturation of 1^st^ generation schizonts (McDougald and Galloway, 1976). Robenidine displayed higher inhibition of replication during continuous treatment rather than with a short pre-treatment during invasion. This is consistent with other findings which revealed a higher antiparasitic effect when the treatment was left in contact with the infected cells for the whole duration of schizogony, confirming the specific efficacy against intracellular development (Arias-Maroto et al., 2022; Felici et al., 2024).

Among the tested botanicals, TEO exhibited a strong invasion-blocking effect in the pre-incubation exposure assay, reducing entry by 40-50%, but also affecting development, with a significant reduction of the replication rate compared to the control. The other compounds showed similar efficacy. These findings agree with previous research reporting that terpenoids such as THY and CAR, major components of TEO and OEO, can rapidly destabilize the parasite membranes and reduce sporozoite infectivity (Felici et al., 2023). However, a stronger effect of botanicals was observed under continuous exposure, especially on replication, highlighting a possible cumulative impact of these compounds as the parasite develop. These findings further support the notion that essential oils interfere with multiple aspects of the intracellular development and replication, including parasite vacuole integrity, oxidative response, and cell immunity, acting as multi-target anticoccidial agents (Muthamilselvan et al., 2016).

Some variability was observed on the inhibition of invasion between both replicates in the continuous exposure experiments. In the first experimental replicate, none of the treatments showed a reduction in invasion, while in the second the invasion inhibition was more marked, likely reflecting the intrinsic variability of these *in vitro* assays (Arias-Maroto et al., 2024). Regardless of this less reliable invasion evaluation, the primary aim and most significant outcome of the continuous exposure experiments was on intracellular replication, which was markedly reduced for all treatments.

The EtNluc-based *in vitro* assay proved to be highly sensitive, reproducible, and well suited for dissecting stage-specific effects of the tested anticoccidial compounds. Unlike fluorescent microscopy or qPCR, this luminescent system offers real-time quantification, enabling higher throughput and lower variability (Vinayak et al., 2015). The correlation between luminescence signal and renowned drug activities validates this approach as a reliable platform for screening both synthetic and natural product. Furthermore, the ability to perform repeated measurements at different time points minimizes the number of parasites required, thereby reducing animal sacrifice and aligning with the 3Rs principle in parasitology research (Russel and Burch, 1959).

## 5 Conclusion

This study reports the successful generation of a NLuc-expressing *Eimeria tenella* strain and demonstrates its application as a sensitive, rapid, and reproducible platform for *in vitro* anticoccidial screening. By distinguishing the effects of treatments applied during short pre-incubation versus continuous exposure, the assay enabled assessment of stage-specific activity against both invasion and intracellular replication. Commercial anticoccidials such as salinomycin and robenidine displayed the expected inhibitory profiles, thereby validating the approach. Importantly, natural compounds including thyme and oregano essential oils and their constituents thymol and carvacrol, significantly impaired parasite development, particularly under continuous exposure, supporting their potential as alternative anticoccidial agents. Together, these findings highlight the value of the NLuc-based assay in advancing discovery pipelines while reducing animal use, and highlight plant-derived compounds as promising candidates for integrated coccidiosis control strategies.  

## Data Availability

The raw data supporting the conclusions of this article will be made available by the authors, without undue reservation.
