# Supplementary material for: Bioluminescence-based in vitro assay for rapid and quantitative anticoccidial screening
Source: Front Cell Infect Microbiol. 2026 Feb 26;16:1773469. doi: 10.3389/fcimb.2026.1773469 (PMC12979480; doi:10.3389/fcimb.2026.1773469)
Supplement: Supplementary file 1 [file DataSheet1.docx]

Supplementary Material

# Supplementary Figures and Tables

**
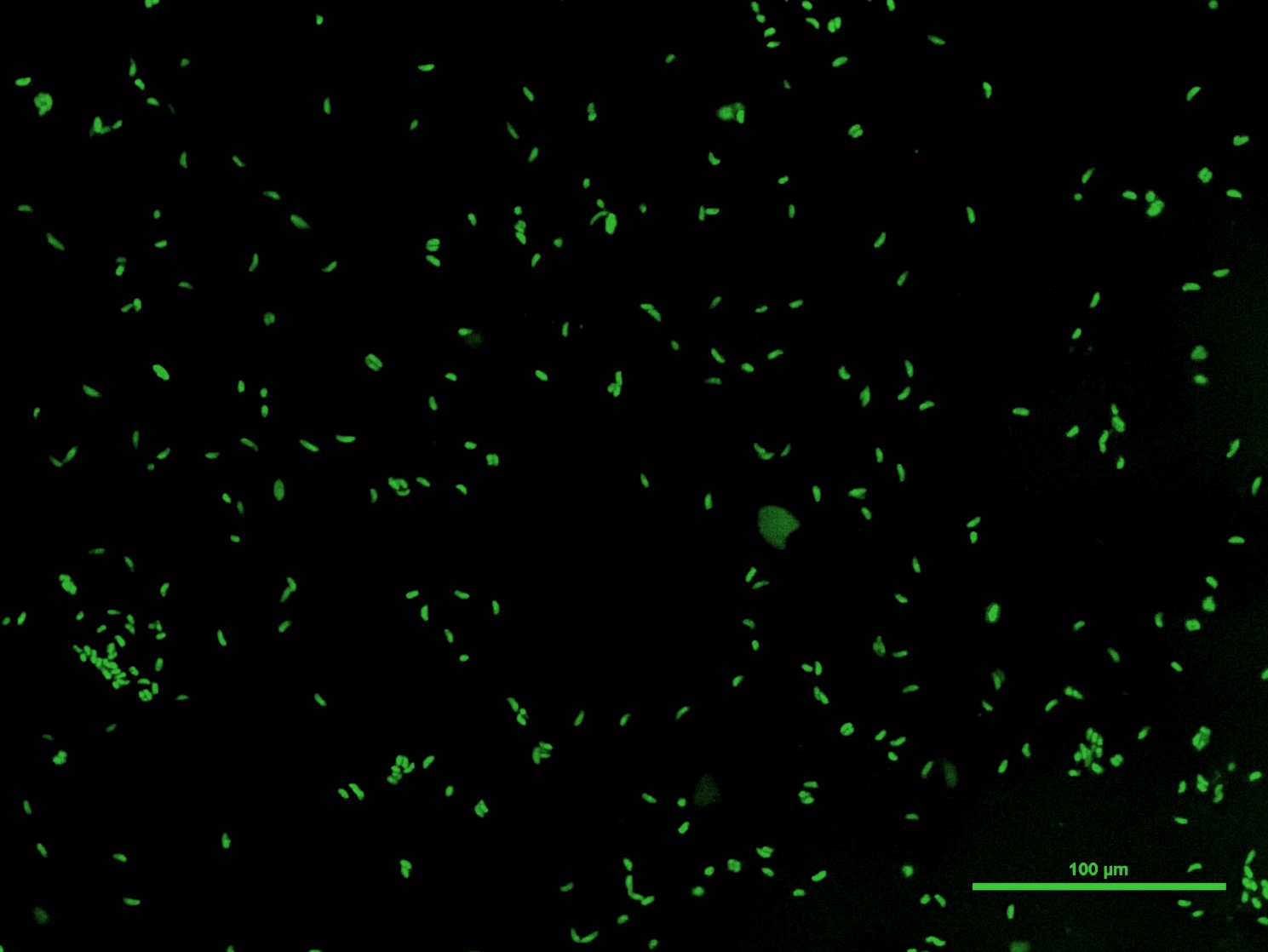
**

**Supplementary Figure 1.** The figure shows MDBK cells infected with stable EtNluc. The transgenic parasites EtNluc exhibited expression of the fluorescent marker mCitrine (green fluorescence).

**Supplementary Table 1.** Evaluation of bioluminescence emission in freshly hatched EtNluc sporozoites and on the reference strain EtWis.

| No. sporozoites | EtNluc (RLU) | EtWis (RLU) |
| --- | --- | --- |
| 1x10^6^ | 76, 998 | 267 |
| 5x10^5^ | 19,063 | 324 |
| 1x10^5^ | 5,185 | 200 |
| 5x10^4^ | 4,563 | 156 |
| 2.5x10^4^ | 3,642 | 101 |
| 1x10^4^ | 1,463 | 97 |
